# Mentor-mentee Relationship: A Win-Win Contract In Graduate Medical Education

**DOI:** 10.7759/cureus.1908

**Published:** 2017-12-05

**Authors:** Hale Z Toklu, Jacklyn C Fuller

**Affiliations:** 1 Graduate Medical Education, HCA/UCF North Florida Division; 2 GME, Ocala Regional Medical Center/ University of Central Florida College of Medicine

**Keywords:** resident training, scholarly activity, mentorship, mentor, advisor, medical resident, graduate medical education, internship and residency, residency, clinical training

## Abstract

Scholarly activities (i.e., the discovery of new knowledge; development of new technologies, methods, materials, or uses; integration of knowledge leading to new understanding) are intended to measure the quality and quantity of dissemination of knowledge. A successful mentorship program is necessary during residency to help residents achieve the six core competencies (patient care, medical knowledge, practice-based learning and improvement, systems-based practice, professionalism, interpersonal and communication skills) required by the Accreditation Council for Graduate Medical Education (ACGME). The role of the mentor in this process is pivotal in the advancement of the residents’ knowledge about evidence-based medicine. With this process, while mentees become more self-regulated, exhibit confidence in their performance, and demonstrate more insight and aptitude in their jobs, mentors also achieve elevated higher self-esteem, enhanced leadership skills, and personal gratification. As such, we may conclude that mentoring is a two-sided relationship; i.e., a 'win-win' style of commitment between the mentor and mentee. Hence, both parties will eventually advance academically, as well as professionally.

## Editorial

Introduction

In Greek mythology, as told in Homer's Odyssey, *Mentor* was a loyal friend and adviser to the King of Ithaca, Odysseus. When Odysseus left for the Trojan War, Mentor raised Odysseus' son, Telemachus, and prepared him to be the king. While doing this, Mentor encouraged Telemachus to take a journey. Athena, the Goddess of Wisdom, took the shape of Mentor and accompanied Telemachus during his journey to Pylos and Sparta [1].

Today, the terms '*advisor*' and '*mentor*' are often used synonymously. Although similar, they refer to different concepts. Advisor is a person who gives advice in a particular field based on their expertise, while a mentor is an experienced and trusted adviser. A few examples of famous mentor-mentee relationships in history are Plato mentor to Aristotle, Aristotle mentor to Alexander the Great, and Sigmund Freud mentor to Carl Jung.

As the moral of the anonymous tale of Rabbit's PhD thesis goes, it could be argued that your mentor matters rather than your thesis subject (*the anecdote is available on http://mfleck.cs.illinois.edu/parable.html*).

Docere is a Latin word first introduced by Cicero in his book De Oratore. The term means to instruct, teach, advice and also refers to the learned person i.e., the scholar. The word doctor originated from 'docere'. Thus, teaching and shaping medical students, residents, and fellows are essential roles of a physician [2]. Even though the distinction between advisor and mentor is not clear in most programs, the difference between the roles of advisor and mentor is of critical importance. As the roles get clear, the expectations and satisfaction rates improve [3].

Scholarly activities are intended to measure the quality and quantity of dissemination of knowledge. The role of mentors in this process is crucial in terms of spreading evidence-based medicine [4].

The metrics may vary among programs and institutions; however, publishing journal articles, presenting in meetings and delivering lectures are commonly accepted scholarly activities [2]. External funding and citations are additional measures of these activities.

When the medical jungle is taken into consideration, guiding medical residents as mentors is crucial. Effective mentorship helps the residents develop the six core competencies (patient care, medical knowledge, practice-based learning, systems-based practice, professionalism, and communication skills) required by Accreditation Council for Graduate Medical Education (ACGME).

Mentoring residents is an inevitable task of a clinician educator in academic medicine. It is a two-sided relationship which increases visibility within the medical community when appropriately established. It is a key factor in helping to promote scholarly activities as well as scholarly productivity as a whole. This 'win-win' style of commitment-mentorship, between the mentor and mentee, will eventually advance both parties academically as well as professionally. In addition to the knowledge and experience gained by both participants, mentees become more self-regulated, exhibit confidence in their performance, and demonstrate more insight and aptitude in their jobs. On the other hand, mentors achieve elevated higher self-esteem, enhanced leadership skills, and personal gratification.

The roles and qualities of the mentor and mentee are summarized in Table 1.

**Table 1:** The roles and qualities of the mentor and mentee

Mentor	Mentee
Active teaching Hearing Pointing to the gold mine Showing the path Probing to raise the bar Being a role model Respect Focus Empathy Devoting time Door opener/ Problem solver Inspires trust Does the right thing Promotes ideas Motivates/Inspires Carves new roads Thinks long term – the end goal Charts new growth	Active learning Listening Digging the gold mine Taking the path Dedication to raise the bar Mirroring the role model Respect Focus Empathy Devoting time Cooperate to solve problems Develops trust Do things right Adapts ideas Motivates/ Inspires Follow the map Meets the expectations Adapts growing

**Figure 1:**
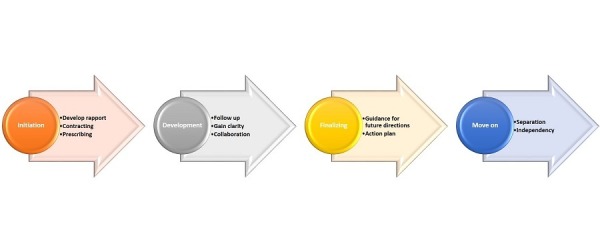
Phases of the relationship between the mentor and mentee

As with other developmental processes, mentoring also has a life cycle which moves from the initiation phase where the mentor and mentee get to know each other and set expectations, to the final phase when the mentoring relationship has achieved its mission, and both participants have mutually agreed to function independently (Figure 1). The length of each phase depends on the interaction and dynamics related to the readiness, enthusiasm, and devotion of both the mentor and the mentee.

Although mentorship has been in existence for centuries, the influx of current data documented in the literature has created a sense of urgency among graduate medical education programs and institutions to establish a system in which residents are paired with official mentors. Several studies showed its effectiveness in integrating clinical, professional, and academic development on an individualized basis [3, 5]. The feedback and guidance are highly appreciated by trainees because of the comprehensive approach to personal interests, skills, and competencies.

It is evident that the success of a resident’s scholarly productivity is highly contingent on the synergy developed between the mentor and mentee. Students’ feedback indicates that a long-term commitment between the mentor and the mentee positively outweighs an individual encounter [5].

Conclusion

The implementation of a successful mentorship program is an effective strategy for creating an environment which will promote scholarly activities among residents in terms of quality and quantity. Both mentors and mentees will significantly benefit from exchanging ideas and sharing knowledge, as well as personal growth and professional development. Additionally, academic institutions will benefit from creating a culture that supports and promotes scholarship via an effective mentorship program.
